# Hyperglycemia induced testicular damage in type 2 diabetes mellitus rats exhibiting microcirculation impairments associated with vascular endothelial growth factor decreased via PI3K/Akt pathway

**DOI:** 10.18632/oncotarget.23915

**Published:** 2018-01-04

**Authors:** Lingli Long, Han Qiu, Bing Cai, Ningning Chen, Xiaofang Lu, Shuhui Zheng, Xiaoxin Ye, Yubin Li

**Affiliations:** ^1^ Translation Medicine Center, The First Affiliated Hospital, Sun Yat-Sen University, Guangzhou, China; ^2^ Department of Medicine, Li Ka Shing Faculty of Medicine, The University of Hong Kong, Hong Kong, China; ^3^ Department of Orthopedics, The Seventh Affiliated Hospital, Sun Yat-sen University, Shenzhen, China; ^4^ Department of Pathology, The First Affiliated Hospital, Sun Yat-Sen University, Guangzhou, China; ^5^ University of New South Wales, Sydney, Australia; ^6^ The Reproductive Center of the First Affiliated Hospital, Sun Yat-Sen University, Guangzhou, China

**Keywords:** vascular endothelial growth factor, diabetes mellitus, testis, PI3K/Akt pathway, rats sertoli cells

## Abstract

As an endocrine disease, type 2 diabetes mellitus (T2DM) can cause testicular damage which induces male infertility. However, the underlying mechanism is still not clear. We prove that T2DM induced testicular microcirculation impairment involves the decrease of VEGF and these actions are regulated by PI3K/Akt pathway. In our study, rats were divided into three groups (n=8): control group, diabetes group and diabetes + VEGF group. Intraperitoneal injection of streptozotocin (STZ, 65mg/Kg, at 9^th^ week) and daily high-fat diet were used to establish T2DM rat model. Serum glucose in diabetes group and diabetes + VEGF group obviously exceeded 13mmol/L after STZ injection. Immunohistochemical studies indicated that VEGF level in diabetes group significantly decreased. In diabetes group, testicular blood velocity and vascular area reduced evaluated by Doppler and FITC. Furthermore, atrophic testicular morphology and increasing apoptosis cells were evaluated by haematoxylin and eosin staining and TUNEL assay. In diabetes + VEGF group, the administration of VEGF (intraperitoneally, 10mg/kg) can significantly alleviated hyperglycemia-induced impairment of testes in above aspects. Finally, we used Western blot to analyze the mechanism of hyperglycemia-induced testicular VEGF decrease. The results indicated that hyperglycemia-induced VEGF decreased is regulated by PI3K/Akt pathway in Rats testicular sertoli cells (RTSCs). Together, we demonstrate that T2DM can reduce testicular VEGF expression, which results in testicular microcirculation impairment, and then induces testicular morphological disarrangement and functional disorder. These actions are triggered by PI3K/Akt pathway. Our findings provide solid evidence for VEGF becoming a therapeutic target in T2DM related male infertility.

## INTRODUCTION

Diabetes mellitus (DM) is a common chronic endocrine metabolic disorder characterized by hyperglycemia with increasing incidence. It is estimated that by 2030, 366 million people will suffer from DM [1, 2]. DM induces many functional and structural syndromes and complications in multiple organs, such as testis, brain, heart and retina. [3–5]. Emerging evidence has shown that diabetes can cause male reproductive dysfunctions [6]. Reduced testicular function was widely observed in animals with hyperglycemia [7]. In human with diabetes, spermatogenesis, sperm count, sperm motility, seminal fluid volume and testosterone levels are lower compared to healthy individuals [7–9]. Since the average age of patients suffered from diabetes-induced reproductive damage is getting younger, finding out the way to protect human reproductive health is in urgent need.

In diabetic patients, impotence, degenerate fertility characterized by abnormal sperm, retrograde ejaculations, and erectile dysfunction have been found. Diabetes induces increasing oxidative stress to the male reproductive system. During diabetes, the balance between the production of reactive oxygen species (ROS) and the ability of deoxidation is disturbed. The superfluous ROS can impair normal structure of cells including proteins and DNA, thus bringing damage to cellular function. Previous study suggested that during diabetes, oxidative stress impaired testicular DNA, survival of reproductive cells and function of spermatogenesis, leading to the male infertility [10, 11]. The most essential damage of diabetes-induced oxidative stress is vasculopathy, which results in diabetic microvascular complications. Therefore, insufficient blood supplement is the main cause of DM-induced testicular dysfunction. A study conducted by Schoeller also indicated that the destroy effect of STZ on testes was induced by the reduction of testosterone level related to diminution of serum insulin levels, because insulin can prevent testicular apoptosis and sexual disorder induced by DM [12]. Many diabetic complications and damage are closely related to the abnormal level of vascular endothelial growth factor (VEGF) in circulation [6]. VEGF, originally known as vascular permeability factor (VPF), is an angiogenic and neurotrophic growth factor activated by insulin and various growth and survival factors [13]. It plays an important role in regulating survival and apoptosis by regulating proliferation of endothelial cells and permeability of the vessel [14]. VEGF participates in the restoration of oxygen and creates new vessels when normal circulation is affected by diabetes-induced hypoxia [15]. VEGF participates in many diabetic processes by regulating redox activities inside body. Importantly, previous studies have shown that VEGF is also very important in maintaining male reproductive function and germ cell homeostasis [16].

Since VEGF plays an important role in both male reproductive health and developmental process of diabetes, the effect of VEGF on diabetic testicular damage is worth discussion. However, currently, the role of VEGF played in testicular damage in diabetic condition is little investigated. The plasma concentration of VEGF in type 2 diabetes mellitus was found higher than normal [17–19]. It is considered that diabetic retinopathy (DR) may be caused by the enhanced level of regional VEGF in diabetes. However, the regional level of VEGF in testes of diabetic rats was lower than normal with increased apoptosis and testicular damage, suggesting that the lack or the reduction of VEGF may be responsible for male dysfunction in diabetes; and VEGF may play a regulative role in testicular damage during diabetes. Also, the underlying mechanism of VEGF's effect may be different from that of other organs [6, 20]. In diabetic condition, little is known about the specific effect of VEGF on testes and the underlying mechanism of VEGF's action.

Previous studies have shown that some cells can resist oxidative stress by enhancing VEGF level, and VEGF/PI3K/Akt pathway is activated in oxidative condition [20, 21]. Meanwhile, VEGF has been proved to be correlated with testicle health. Therefore, we hypothesize that T2DM induced testicular damage as a results of down regulation of testicular VEGF level, which was triggered by PI3K/Akt pathway. Our study can provide evidences for the use of VEGF in the pathogenesis and prevention of male infertility in T2DM.

## RESULTS

### General feature in T2DM rat model

**Figure 1:**
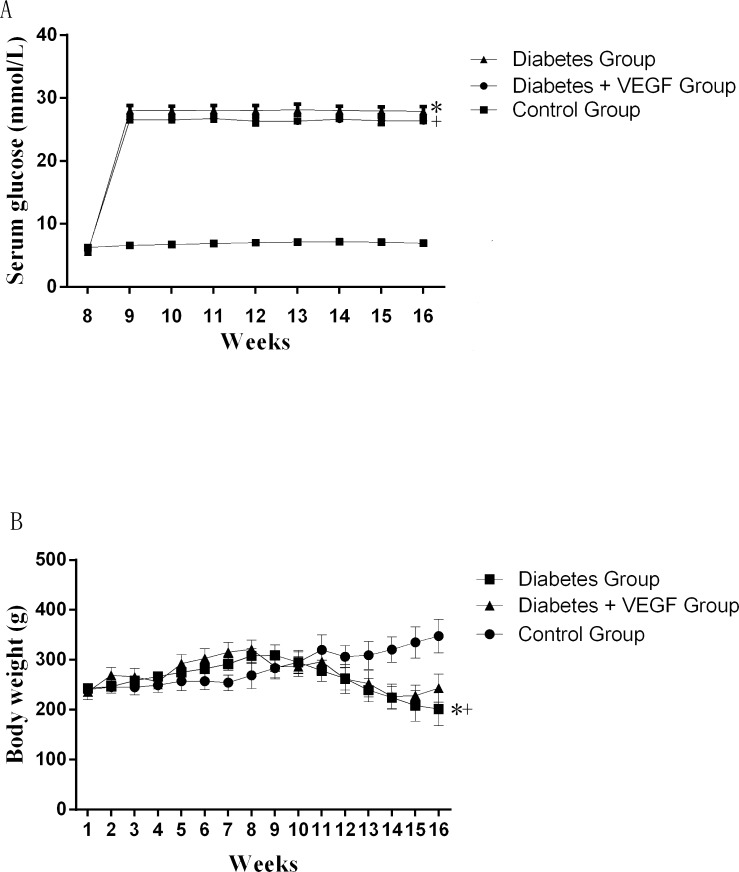
The change of serum glucose and body weights Serum glucose **(A)** and body weights **(B)** in diabetes rats compared to control group and diabetes +VEGF group. Diabetes group and diabetes + VEGF group were intraperitoneally injected with STZ at week8. From week 9, diabetes + VEGF group was daily treatment with VEGF. ^+*^P < 0.05 compared with control group, +p > 0.05, compared with diabetes group (n=8).

In order to characterize the T2DM rats used in our study, body weight from week 1 to week 16 and blood glucose from week 8 to week 16 were evaluated (Figure 1). Blood glucose of rats obviously exceeded 13mmol/L after the injection of STZ (at week 8) in Diabetes group and Diabetes + VEGF group, but statistical study did not show significant difference between these two groups (*P* > 0.05). Blood glucose of control group fluctuated in the normal range. Body weight of Diabetes group and Diabetes + VEGF group increased from week 1 to week 8, but after the injection of STZ at week 9, it displayed a downtrend. Statistical study also did not show significant difference between these two groups (*P* > 0.05). Body weight of control group kept an uptrend from week 1 to week 16. Table 1 shows the testicular weight and epididymal weight of all groups at week 16.

**Table 1:** Testicular weight and epididymal weight of all groups

Weight (g)	Control group	Diabetic group	Diabetes +VEGF group
Testes	2.04±0.32	1.13±0.12^*^	1.67±0.22^#^
Epididymis	0.72±0.14	0.25±0.06^*^	0.51±0.06^#^

a. ^*^Significantly different from control group (*p* < 0.05).

b. ^#^Significantly different from diabetes group (*p* < 0.05).

c. Values are expressed as mean ± standard error of the mean (SEM).

### The alterations of VEGF expression in testes

**Figure 2:**
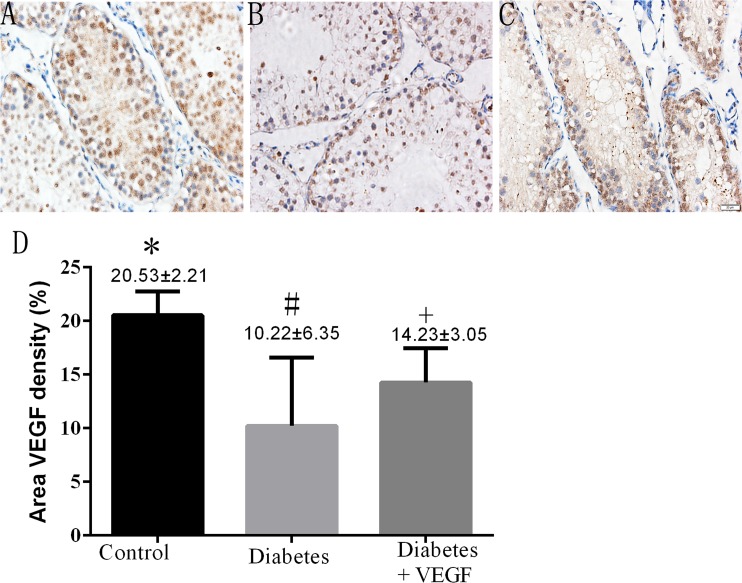
Immunostaining of testicular VEGF in all groups **(A)** control group, **(B)** diabetes group, **(C)** diabetes + VEGF group. DAB was used as background staining. VEGF density (% of area) was determined using Image-Pro Plus to calculate the total area of positive cells. Data were presented as means ± SEM **(D)**, three fields per section and five sections from each testis, n = 8 rats for each group.

To investigate the different expression levels of testicular VEGF among control group, diabetes group and diabetes +VEGF group, immunohistochemical study was performed on representative testis slices from each group. Cytoplasmic staining of VEGF in the photographs demonstrated an approximate 50% reduced expression of VEGF in diabetes group compared to control group, while the diabetic +VEGF rats showed a higher expression level of VEGF compared to diabetes group (Figure 2A, *p* < 0.05). Therefore, diabetes-induced microcirculatory damage is possibly related to decreased expression of VEGF.

### Evaluation of testicular microcirculation by doppler and FITC

**Figure 3:**
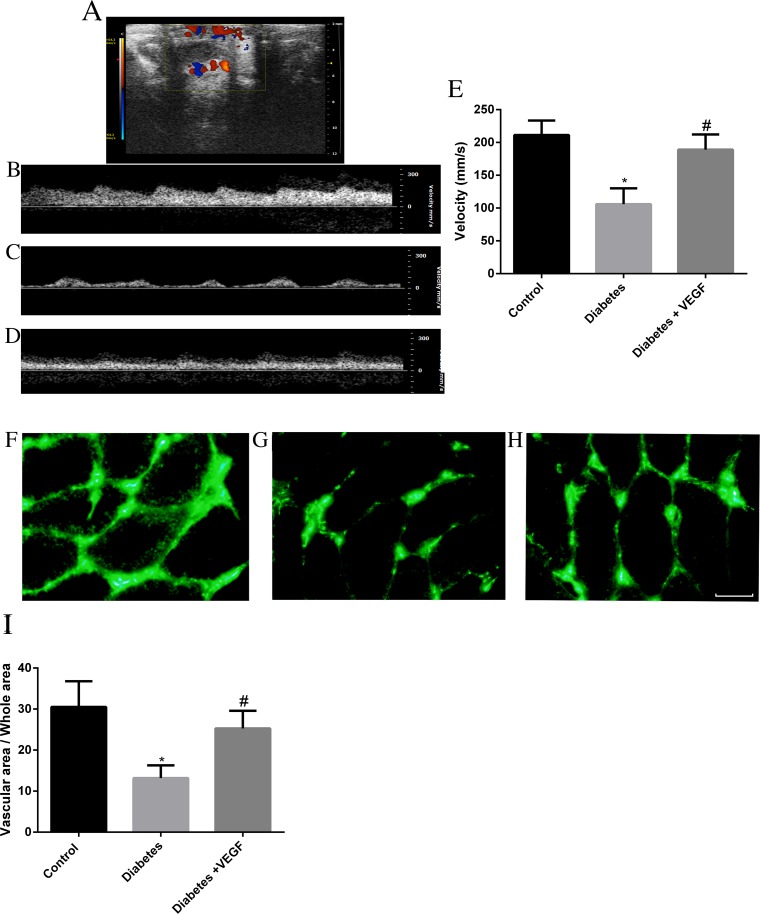
Color Doppler sonography analysis and FITC-dextran label was performed in all groups **(A)** Color Doppler ultrasound image from a diabetes + VEGF rat testis indicated localization of testicular blood vessel. Testicular blood flow rate in control group **(B)**, diabetes group **(C)**, and diabetes + VEGF groups **(D)** were showed by spectral analysis. Quantitative evaluation of velocity was showed in **(E)**. FITC-dextran was used to label vascular area. The rats from control group **(F)**, diabetes group **(G)**, and diabetes + VEGF groups **(H)** were perfused with fluorescein isothiocyanate (FITC)-dextran (green) from tail vein. Slides were prepared for each group and analyzed by fluorescence microscopy (200×). **(I)** Quantification of vascular areas in the testes of rats. Three fields per section and five sections from each testis. Data presented as means ± SEM, n = 8 rats for each group. ^#^*p* < 0.05, compared to control group and diabetes group; ^*^p <0.05, compared to control group and diabetes + VEGF group.

**Figure 4:**
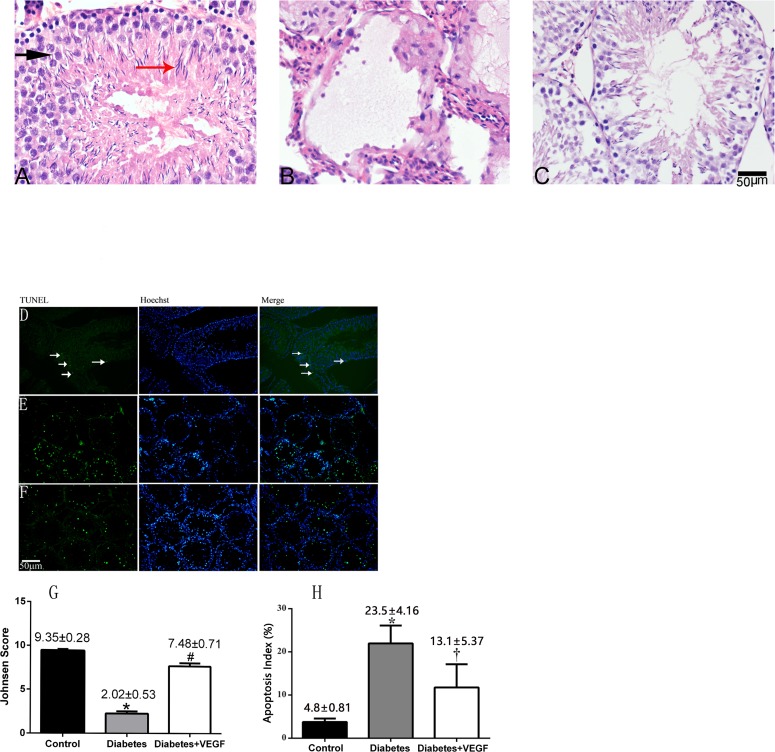
The changes of testicular morphology and cells apoptosis in all groups Testicular morphology in diabetes rats and diabetes + VEGF rats compared to control at 16 weeks of age **(A-C)**. (A) H&E staining in control rats (400×), spermatids (black arrow) and spermatozoa (red arrow) was indicated. (B) H&E staining in diabetic rat (400×). (C) H&E staining in diabetes + VEGF rat (400×). Johnsen Score of rats in testicular sections were present below it **(G)** ^*^p < 0.05 compared to the control group, ^*^p < 0.05 compared to Diabetes + VEGF group, #p < 0.05 compared to Control group. **(D)-(F)** TUNEL staining of testicular sections from control, diabetes, and diabetes + VEGF rats. TUNEL-positive cells (white arrows) in representative images were showed in green (200×). Hoechst was used as cell nucleus staining. The percentage of apoptotic cells was quantified in **(H)** and calculated following the formula Apoptosis index = apoptotic cells / (apoptotic cells + normal cells). Data is presented as means ± SEM, three fields per section and five sections from each testis. n = 8 rat for each group. ^*^Significantly different from control group (*p* < 0.05); ^†^significantly different from diabetes group and control group (^†^*p* versus control group < 0.05; ^†^*p* versus diabetes group < 0.05).

We used color-coded Doppler ultrasound to measure testicular blood flow rate of rats in control group, diabetes group and diabetes + VEGF group. During real-time scanning, arterial pulsation of blood in this vessel was easily observed by Color-coded Doppler ultrasound images (Figure 3A). Velocity was reduced from 211.25±22.1mm/s in control group (Figure 3B&3H) to 105.87±24.2mm/s in diabetes group (Figure 3C&3H, *P* <0.05). Compared to diabetes group, velocity of diabetes + VEGF group increased up to 189.15±23.1mm/s (Figure 3D&3H, ^*^p ≤ 0.05, compared to control group; #*p* ≤ 0.05, compared to diabetes group). Generally, we used velocity to represent blood flow rate. The reduction of velocity was attributed to the hyperglycemia-induced microcirculatory damage. In order to confirm the microcirculatory changes, testes were perfused with fluorescein isothiocyanate (FITC)-dextran (green). The result revealed that hyperglycemia markedly reduced the vascular area in testes (Figure 3F), and administration of VEGF can increase the vascular area (Figure 3G). The ratio of vascular area and whole area were showed in Figure 4I. The percentage in control group, diabetes group and diabetes +VEGF group was 30.50±6.3%, 13.2±3.1% and 25.3±4.3% respectively (Figure 4I). These results suggested that hyperglycemia could lead to testicular ischemia, which decreased the blood velocity. Interestingly, the administration of VEGF availably improved microcirculation.

### Changes of testicular morphology and apoptosis

The above studies showed that hyperglycemia decreased the expression of VEGF, resulting in microcirculatory damages. Therefore, we used H&E staining and TUNEL staining to evaluate whether the microcirculatory damages resulted in morphological destruction and cellular apoptosis in testes. In these studies, rats of control group had normal testicular structure and regular seminiferous tubular morphology with a great number of spermatids (black arrow) and spermatozoa (red arrow, Figure 4A). In the diabetes group, atrophic seminiferous tubules with seldom germ cells while some sertoli cells were found. The seminiferous tubules were disrupted (Figure 4B). With VEGF treatment, the structure of testes was more completive than that of diabetes group, but abnormal spermatozoa and many spermatids presented (Figure 4C). Evaluation of spermatogenesis by the Johnsen score indicated the testicular condition of all groups. The lower Johnsen score represented more serious testicular damages. The mean Johnsen score in control group was 9.35±0.28, which decreased to 2.02 ± 0.53 in diabetes group and rose to 7.48± 0.71 in diabetes + VEGF group. The diabetic rats showed more failure in spermatogenes, while VEGF could reverse these hyperglycemia-induced failures. Our TUNEL experiments also revealed numerous apoptotic cells of testes in diabetes group (Figure 4E) and VEGF effectively prevented hyperglycemia-induced cell death (Figure 4F). The apoptosis index was 4.8%, 23.5%, and 13.1% in control group, diabetes group, and diabetes + VEGF group, respectively (Figure 4G). These data suggested that in the response to hyperglycemia-induced microcirculatory damages, testicular structure collapsed and number of apoptotic cells was increased.

### Sperm analysis

**Figure 5:**
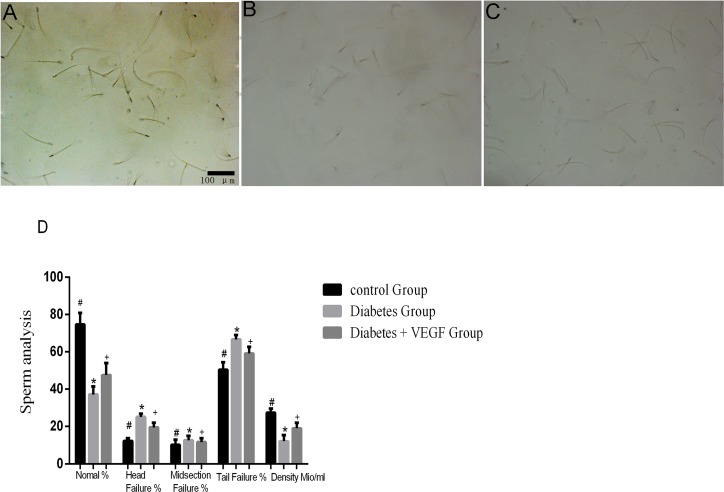
Sperms conditions in all groups Sperms from control group **(A)**, diabetes group **(B)** and diabetes + VEGF group **(C)** in 16-week age. Sperms were squeezed out and observed by microscope (200×). Sperm analysis were present in **(D)** ^*^Significantly different from control group (*p* < 0.05); ^+^significantly different from diabetes group and control group (^+^*p* versus control group < 0.05; ^+^*p* versus diabetes group < 0.05).

In order to assess the relation between sperm failures and VEGF, we released sperms into petri dishes and observed them under microscope. The density of sperms in diabetes group was the lowest one. The results also showed that the percentage of failure sperms (head, midsection and tail) in diabetes group was higher than control groups, while VEGF could reverse the hyperglycemia-induced failure of sperms. These results indicated that VEGF had a protective effect on hyperglycemia-induced sperms failure and decrease in quantity (Figure 5).

### Diabetes-induced VEGF decreased through PI3K/Akt pathway

**Figure 6:**
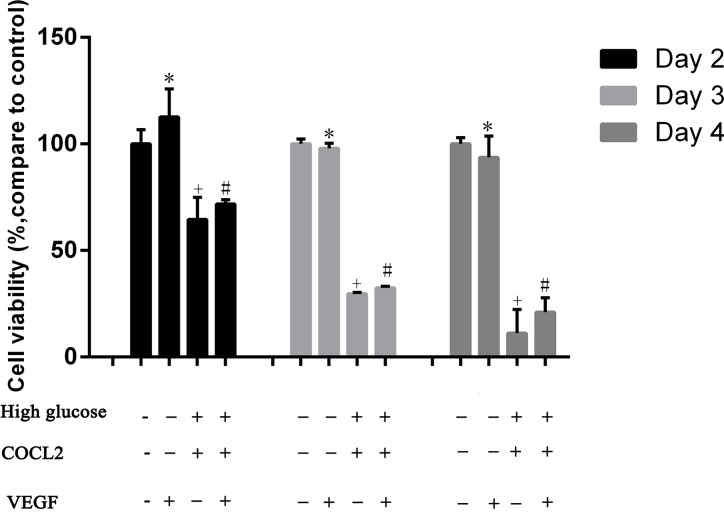
Cell viability was measured by CCK8 Rats sertoli cells (RTSCs) were divided into four groups. RTSCs in control groupVEGF group, Diabetes group and VEGF + Diabetes. Control group and VEGF group were treatment with normal medium. Diabetes group and VEGF + Diabetes group were cultured in high glucose medium with CoCl_2_. The cell viability of control group was viewed as the baseline (100%), and other groups compared with the control to show their cell viability in different culture environments. ^*^P<0.05, ^+^P<0.05 *and* ^#^*P<0.05* compared to control group; ^+^*P<0.05* compared to VEGF + CoCl_2_ group.

The insulin-regulated activation of PI3K/Akt is important to regulate glucose disposal and other metabolic adaptations [22]. Previous studies pointed out that PI3K/AKT has the ability to control most cells survival and angiogenesis. Therefore, we supposed that in T2DM rats, hyperglycemia-induced testicular lesions were regulated by PI3K/Akt/VEGF pathway. We cultured Rats sertoli cells (RTSCs) in high glucose medium with pharmaceuticals cobalt chloride (CoCl_2_) to imitate diabetic environment (high glucose and hypoxia). Firstly, our results showed that compared to the control group (RTSCs in the common medium), the viability of RTSCs in high glucose medium with CoCl_2_ decreased, while the administration of VEGF improved their viability (Figure 6A). Secondly, our biochemical studies proved that PI3K/Akt pathway was involved in diabetes-induced VEGF reduction. We found that in the diabetic environment, the inhibition of p-PI3K/p-Akt (phosphorylation-PI3K/Akt) was triggered, and then the protein of VEGF was down regulated. Similarly, inhibiting PI3K/Akt pathway with LY294002 also blocked VEGF activation. In light of these findings and the previous publications which pointed an angiogenic role of PI3K/Akt pathway, we suggested that inactivation of VEGF in response to diabetes was similar with inhibiting PI3K/Akt pathway by LY294002 (Figure 6B, 6C&6D). Moreover, compared with CoCl_2_ + high glucose stimulated cells, VEGF treatment could active PI3K/Akt (P<0.05, compared to CoCl_2_ group), which indicated that VEGF can interact with PI3K/Akt pathway and thus play a protective role in diabetic testes.

## DISCUSSION

Diabetes mellitus (DM), a pathological condition of hyperglycemia, can cause damage in multiple organs and systems, including male reproductive system dysfunction. Type 2 diabetes mellitus (T2DM) is characterized by absent responsiveness of body tissues to insulin, namely, insulin resistance. It is easily to induce long-term complications which lead to destroy in small blood vessels, include damage to eyes, nerves and testes [13, 23, 24]. VEGF is an important protein secreted by cells and participates in angiogenesis and vasculogenesis. It also involves in restoring the oxygen supply during inadequate blood circulation by regulating endothelial metabolism. Jesmin et al. found that diabetic cardiomyopathy could be prevented through restoration of VEGF, which improved microcirculation in coronary. Also, through up-regulating VEGF, erectile dysfunction can be prevented [25]. VEGF is a potential regulator for diabetes-induced vascular dysfunction and complications. Therefore, our study discussed about the VEGF's specific effect on microcirculation in diabetic testes. Although it has been demonstrated that VEGF is essential for regulating circulation especially regional microcirculation, little is discussed about the VEGF's specific effect and its mechanism on microcirculation in diabetic testes.

Combination of high-fat diet-fed and low-dose STZ-treated rat is a proved model for T2DM [26, 27]. We measured the increased serum glucose of the rats to confirm the successful establishment of T2DM model (Figure 1A). In our results, the body weight of diabetic rats apparently decreased (Figure 1), which is one of the symptoms of diabetes mellitus emerging when serum glucose increase, since hyperglycemia induce the obvious loss of calories, muscle and adipose tissue due to exhaustion of protein [12]. At the same time, we can find a decrease of testes and epididymis in diabetic rats (Figure 1B), indicating that reproductive organs are sensitive to hyperglycemia. The treatments of VEGF only reverse the weight loss of testes and epididymis (Table 1), suggesting that the mechanism of hyperglycemia-induced reproductive dysfunction associated with VEGF level, but VEGF can not affect insulin secretion, which is the main factor to control diabetic rats body weight [28]. Therefore the mechanism of VEGF and diabetes-induced reproductive dysfunction is worth further studying.

Our previous research showed that in diabetic state, microcirculation and hemodynamics were impaired by hyperglycemia, which further brought oxidative stress to testes [29]. Evidence suggested that VEGF plays an important regulative role in testes by maintaining normal blood supply in regional circulation. Also, by triggering vasculogenesis, VEGF participates in the occurrence and development process in diabetes and diabetic complications. In this study, we first time investigated VEGF's effect on diabetes-induced testicular damage and underlying mechanism in molecular level. Our study demonstrated hyperglycemia induced reduction of VEGF in testes, which brought about impaired testicular microcirculation, including decreased blood velocity and vascular area. Insufficient blood flow further resulted in testicular structure and functional damage, e.g. testicular morphological collapse (Figure 4A, 4B, 4C), increased cells apoptosis (Figure 4D, 4E, 4F, 4G) and sperm abnormalities (Figure 5). Meanwhile, the treatment of VEGF on diabetic rats alleviated the hyperglycemia-induced impairment of testes, consistent with previous study in bovine testes [30, 31]. Regional microcirculation was obviously ameliorated. We confirmed our results by *in vitro* study in RTSCs cells (Figure 6), which clearly demonstrated that hyperglycemia-induced testicular insufficient VEGF was regulated by PI3K/Akt pathway, and inhibition of VEGF can down-regulate PI3K/Akt pathway. Our *in vivo* and *vitro* experiments indicated that VEGF plays a protective role in diabetic testicular damage through PI3K/Akt pathway. This is a new opinion about diabetes-induced reproductive disorder, which can give us a perspective to improve the fertility of male diabetic patients by exogenously added VEGF or VEGF genetic therapy.

**Figure 7:**
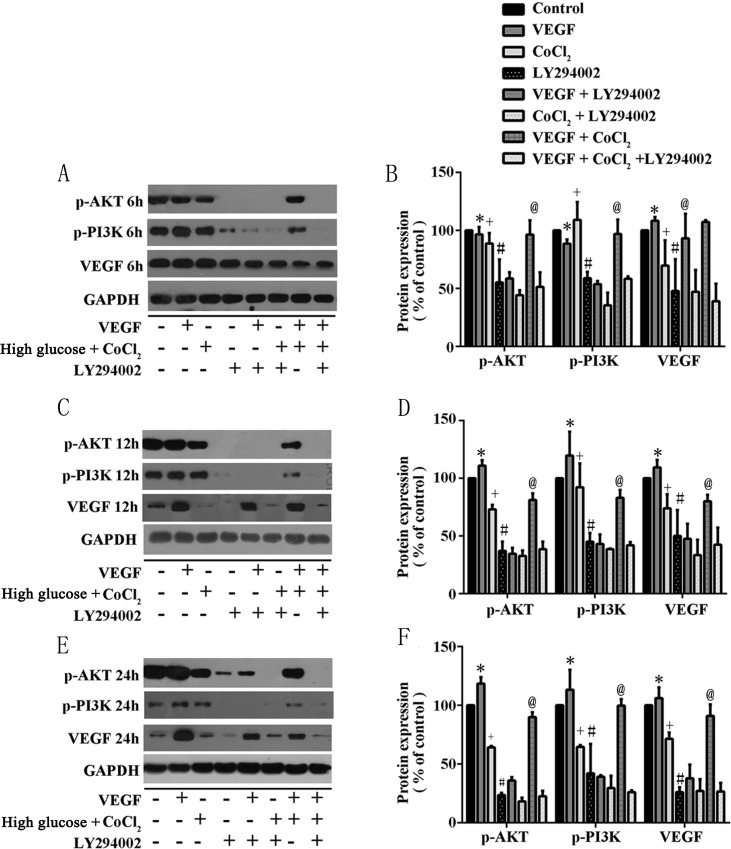
In RTSCs, the production of VEGF interacts with PI3K/Akt pathway under the simulation environment of diabetes RTSCs in the control group were treated with normal glucose medium, while others were treated in different conditions as described in methods. RTSCs in **(A)**, **(C)** and **(E)** were respectively stimulated in 6h, 12h and 24h, and subjected to western blot to analysis with antibodies to VEGF, phospho-Akt, and phospho-PI3K. Bar graphs in **(B)**, **(D)** and **(F)** present quantitative difference in expression of VEGF. Data were presented as means ± SEM. ^*^P<0.05, ^+^P<0.05 *and*^#^P<0.05 compared to control group; ^@^*P<0.05,* compared to CoCl_2_ group.

As we know, VEGF has important regulative effects on vascular metabolism in both healthy and pathologic condition, especially under hypoxia and ischemia environment [16, 32, 33], but existing studies have inconsistent opinions about the effects of VEGF in the diabetic complications. It could be either high despite reduced neoangiogenesis effect, or low, or no change, as in the study by Broderick et al [34]. This depends on the duration and severity of diabetes or to the difference in downstream signaling of VEGF, e.g.: VEGF signaling can be enhanced and then induces angiogenesis effect as endothelial cells response to diabetic ischemia in retina. However, opposite results indicated that in the diabetic myocardium, VEGF has been shown to be decreased that partly brought about anti-angiogenesis and microcirculatory disorder. Furthermore, it has been indicated that diabetes induces the angiogenic paradox: it is related to both increased VEGF in diabetic retinopathy and chronic trauma, or decreased VEGF in arteriogenesis and impaired collateral vessel growth angiogenesis [35–40]. Our study indicated that testicular VEGF was lower expressed in both *in vivo* testes of T2DM rats and RTSCs, consistent with the study conducted by Sisman and his team [5, 6]. The mechanisms of our results involve the inhibitions of PI3K/Akt. Under pathological circumstance, inhibited PI3K/Akt signaling could abrogate the production of VEGF. Akt plays an important regulative role in VEGF expression. Therefore, once PI3K/Akt pathway is blocked, VEGF level will also decrease [41–43]. Tchaikovski et al. reported that VEGF receptor's angiogenic-related signaling response inside body was attenuated in T2DM. PI3K/Akt signal transduction was impaired, further leading to the dysfunction of VEGF [43]. Especially, diabetes-induced testicular damage generally occurs later than other diabetic complications [44]. Our results supported these researches by indicating that the attenuation of Akt and VEGF is in a time-dependent manner. The longer hyperglycemia existed, the lower Akt and VEGF expressed (Figure 6). Therefore, PI3K/Akt signaling is severely inhibited in the diabetic testes, and VEGF expression obviously reduced because of long duration hyperglycemia-induced inactive PI3K/Akt. Moreover, previous studies pay attention on diabetes-induced VEGF high expression in endothelial cells, but our study discussed the diabetes-induced VEGF decreased in RTSCs. Testicular sertoli cells are the primary cells to differentiate in the testes, and they produce greatest abundance VEGF throughout life to trigger endothelial cell migration and proliferation [41, 42, 45]. Therefore, our results implied that hyperglycemia destroyed RTSCs viability (Figure 6), and then the production of VEGF was diminished (Figure 7). In summary, our research indicated that hyperglycemia-induced VEGF decrease in testes related to two mechanisms. Firstly, long-term hyperglycemia inhibits PI3K/Akt pathway which further results in VEGF decreased. Secondly, hyperglycemia reduced the testicular VEGF production by attenuating RTSCs viability.

Importantly, not only Akt participates in the regulation of VEGF expression, VEGF can also activate PI3K/Akt signal pathway. In present study, we confirm this point by showing that under the presence of hyperglycemia and CoCl_2_, administration of VEGF can activate PI3K/Akt phosphorylation (Figure 7). Previous study indicated that activation of Akt by VEGF stimulates endothelial cells proliferation, migration, and apoptosis [16, 46]. Therefore, inhibition of PI3K/Akt induced by the loss of VEGF accounts for attenuated angiogenesis [47, 48]. In diabetes, the interaction between VEGF and Akt signaling and their synchronous deterioration can result in obvious testicular microcirculation damage [49]. Generally, microcirculation dysfunction mainly contains two aspects: blood stream and blood vessels. The dysfunction of blood stream includes the disappearance of spontaneous vasomotion and decrease of vascular area or density. The dysfunction of vessels includes increase of vascular viscosity, decrease of blood velocity, and stasis of flow [50–53]. In our results, we found that both testicular blood velocity and vascular area decreased as a result of hyperglycemia-induced VEGF decrease (Figure 3), indicating that in diabetes, restrained expression of VEGF can indeed cause dysfunction of testicular microcirculation. Microcirculation dysfunction can influence the vascular trophicity, remodel the vessels and cause the reduction of nutritional perfusion. This will not only result in the hypoxia- and ischemia-related damage of testicular cells, but also restrain the elimination of metabolic waste. Therefore, cell apoptosis is the most common injury induced by microcirculation dysfunction. All of these influences can further disturb the homeostasis of testicular environment, cell-tissue interaction and substance-interchange, and cause cell apoptosis [54–58]. In the testes of T2DM rats, much more apoptotic cells appeared, accompanied by abnormal histomorphology (Figure 4). In our study, diabetic testicular tissue got a lower histomorphology score than control, associated with high scale of TUNEL-positive cells (Figure 4). Meanwhile, the percentage of sperm failure was higher than normal (Figure 5), suggesting that testes from T2DM rats suffered from morphological and functional damages. Apoptosis of testicular cells and dysmorphia sperm cells exert a negative influence on conception processes. For example, as an important step for the formation of zygote, the degradation of zona pellucid of ovum needs enzyme from numerous sperm cells, which could not be effectively performed by head failure sperms and low density of semen, and then the failure in the fusion between sperm cell and ovum finally lead to infertility [59, 60]. Our results point out that long-term hyperglycemia triggered the interaction between VEGF and PI3k/Akt signaling in testes of T2DM rats, which destroyed testicular microcirculation, and then further results in morphological damage, cell apoptosis and failure sperms. The reduction of quality and quantity of spermatogenic cells and sperms can both cause infertility. Interestingly, in diabetic testes, is VEGF expression attenuated in protein, mRNA or genetic level? And how does VEGF interact with other hormone, cytokines and factors in the diabetic testes? Therefore, the underlying mechanism is still very complex and needs further research.

**Figure 8:**
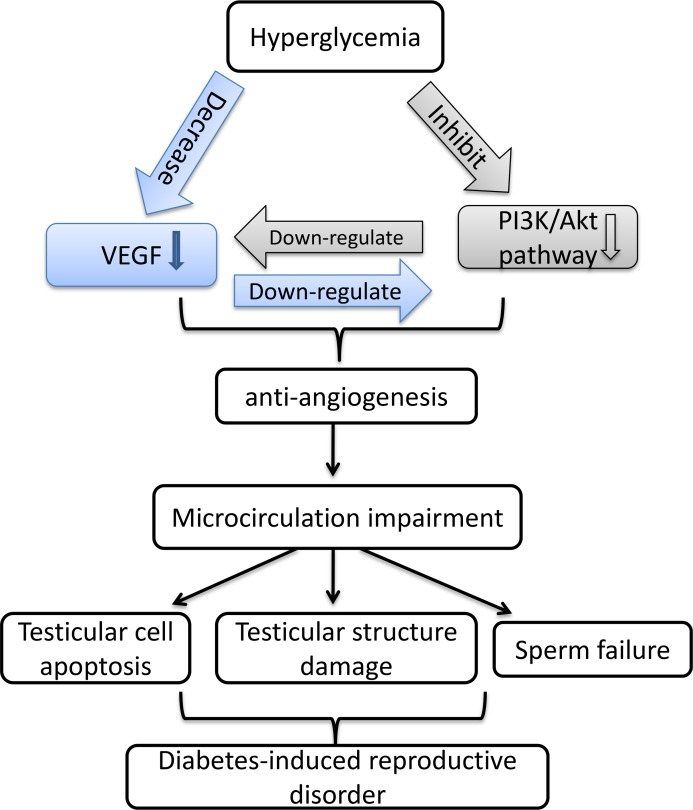
Schematic diagram for the mechanism by which VEGF interact with PI3K/Akt pathway to regulate testicular damage in diabetic rat

In conclusion, our research focused on the T2DM-induced reproductive disorder and its mechanism. We demonstrated that in T2DM rats, long-term hyperglycemia induced testicular microcirculation impairment regulated by the decrease of VEGF, which results in testicular cells apoptosis and abnormal sperms. Mechanistically, our experiments *in vitro* indicated that the decrease of testicular VEGF is related to the damage of rat sertoli cells and inactivation of PI3K/Akt path way triggered by long-term hyperglycemia (Figure 8). Our findings are great importance in considering future studies supporting application of VEGF as a therapeutic target toward T2DM-induced male infertility.

## MATERIALS AND METHODS

### Experimental animal

All animal protocols were approved by China Council on Animal care and Sun Yet-San University Committee and have therefore been performed in accordance with the ethical standards laid down in the 1964 Declaration of Helsinki and its later amendments. Adult male rats weighing 200-250g were obtained from animal facility of Sun Yat-Sen University and housed in stainless steel cages, and kept at a 21°C±4°C temperature, humidity of 40–65% with a 12-hour light/dark cycle. They had access to tap water and commercial chow ad libitum. Rats were divided into three groups (grouping method: random number table): control group (n = 8), diabetes group (n= 8), and diabetes + VEGF group (n= 8). Free access to food and drink water. Normal food (Animal Facility of Sun Yat-Sen University, Guangzhou, China) was given to the control group. High-fat diet (HFD, Guangdong Medical Laboratory Animal Center, Guangzhou, China), containing 30% fat, 15% protein, and 55% carbohydrate, was used in diabetes group and diabetes +VEGF group to induce insulin resistance. After week 9, a single dose of streptozotocin (STZ, Sigma-Aldrich, MO, USA, S0130; 65mg/Kg) dissolved in 0.1M citrate-phosphate buffer was freshly prepared and intraperitoneally administered into diabetes group and diabetes + VEGF group to induce the type II diabetes mellitus (HFD feeding was continued). The dose of STZ and SCU was according to previous published work [61, 62]. The same volume of 0.1M citrate-phosphate buffer was used as vehicle control for the control group. After one week of the injection of STZ, rats in diabetes + VEGF group received VEGF intraperitoneally (10mg/kg; Sigma, USA) everyday for 2 months. The same volume of phosphate buffer was used as vehicle control for control group and diabetes group. Blood was collected from three group rats via the tail-vein and blood glucose was evaluated using automated blood glucose analyzer (Roche, Model GC, Switzerland). Blood glucose level higher than 13mmol/L was considered as type II diabetes mellitus (T2DM). At the end of the experiment, rats were sacrificed by cervical dislocation under intraperitoneal ketamine/xylazine anaesthesia.

### TUNEL staining to evaluate apoptotic cells

We used TUNEL assay with the In Situ Cell Death Detection Kit, POD (Roche, Basel, Switzerland) to evaluate testicular apoptosis, following the manufacturer's instructions. Testicular tissue was fixed in 4% paraformaldehyde, embedded in paraffin, and then sectioned at 5 μm. Briefly, slides were dewaxed and rehydrated in xylene and ethanol followed by incubation with proteinase K working solution at 37°C for 20min. After rinsing with PBS, the samples reacted with TUNEL reaction mixture (50 μL) at 37°C for 1 hour followed by rinsing the slides with phosphate-buffered saline to stop the reaction. Apoptotic cells were analyzed by randomly counting the TUNEL positive cells from sixty cross-sections of seminiferous tubule/slide under the fluorescence microscope at ×200 magnification (Olympus, TH4-200, Tokyo, Japan). Results were quantitative analysis as TUNEL positive cells per 100 cells (Image-Pro Plus 6.0).

### Sperm analysis

The fresh epididymides from all group rats were separated and transferred into a Petri dishes containing 1 ml of high saline bicarbonate (HSB) buffer and milked by forceps to release stored sperm, and then sperm were incubated at 37°C. In order to observe sperm morphology and assessment sperm condition, sperm counting was assayed manually in white blood cell chambers under light microscopy (Olympus, TH4-200, Tokyo, Japan). Finally, data were expressed as the percentage of normal sperm. We followed WHO criteria to evaluate sperm morphology (World Health Organization, 2010).

### Haematoxylin and eosin staining

The testicular samples from each group were fixed in 10% paraformaldehyde, and then these specimens were embedded in paraffin. Five sections from each sample were cut consecutively from paraffin-embedded block. After dewaxing in xylene and dehydrating in graded concentration of alcohol, sections were stained by haematoxylin and eosin (H&E) to evaluate testicular morphology. The results were observed under light microscope at ×400 magnification.

### Immunohistochemical studies

Immunohistochemistry was performed according to the manufacturer's instructions. The slides were prepared as same as TUNEL assay. We immersed slides in citrate buffer, and heated them in a microwave oven for 15min. We used 3% H_2_O_2_ solution in methanol to block Endogenous peroxidase for 20min. After three times washing with TBS, the slides reacted with anti-VEGF primary antibody (Abcam, Britain, dilution 1 : 200) and then reacted with anti-rabbit secondary antibody at 37°C for 30mins followed by three washes with PBS, respectively. Diaminobenzidine (DAB Kit; Beijing Zhongshan Biotechnology Co., China) was used to visualize immunoreactive proteins. The protein levels were detected using Polink-2 Plus IHC Detection System (Beijing Zhongshan Biotechnology Co., Beijing, China) after staining with diaminobenzidine (DAB Kit; Beijing Zhongshan Biotechnology Co., China) to visualize immunoreactive proteins following the manufacture instruction. Diaminobenzidine (DAB) was added to the slides for 3min as a chromogen. Followed by rinsing in running tap water, slides were counterstained with hematoxylin (blue). Finally, slides were dehydrated in absolute ethanol and mounted. All of them were observed under the light microscope at ×400 magnification (Olympus, TH4-200, Tokyo, Japan).

### FITC-dextran label testicular vascularization

Rats from three groups were deeply anesthetized with sodium thiopental and then perfused via the left ventricle with 1 mL of 50 mg/mL FITC-dextran (Sigma-Aldrich, St. Louis, MO) or 1 mL of 2% direct blue dye (Evans blue; Sigma-Aldrich). The rats were euthanized, and testes mounts were prepared after being fixed in 4% paraformal- dehyde for 30 minutes. We used a fluorescence microscope to take images (Olympus, TH4-200, Tokyo, Japan). The testicular vascular areas were quantified using image processing and image analysis software (Image-Pro Plus; Media Cybernetics, Silver Spring, MD) according to a previous protocol [63]. Briefly, the vascular areas on each slide were selected, and the numbers of pixels comprising the selected areas were counted. The relative testicular vascular areas and whole areas were calculated by comparing the number of pixels in the affected areas with the total number of pixels in the testes.

### Cell culture and cell treatment

Rats sertoli cells (RTSCs) were bought from the American type culture collection (ATCC, Manassas, VA). These cells were cultured in DMEM, High glucose (Gibco BRL, Grand Island, NY, USA) supplemented with 10% FBS (fetal bovine serum; Gibco BRL, GrandIsland, NY, USA), 100 U/ml penicillin and 100 U/ml streptomycin. All cells were stored at 37°C in a CO2 incubator with a controlled humidified atmospherecomposed of 95% air and 5% CO2. We added CoCl_2_ (200 μm/L) into the high glucose medium to imitate the diabetic environment. Before western blot experiments, some cells were treatment with LY294002 (10μm) to block PI3K/AKT pathway, and some were added VEGF (25ng/ml) to culture.

### Cell viability

Cell viability was evaluated by cell counting kit-8 (CCK-8; Dojindo Molecular Technologies Inc., Kumamoto, Japan). Briefly, RTSCs were transfer into 96-well plate (approximately 5000 cells/well), and were further incubated for 24 hours 37°C. After replacement with fresh medium, VEGF-A (25ng/ml) [64], CoCl_2_ (200 μm/L) [65], VEGF and CoCl_2_ were added to each well respectively for another 2 days, 3days and 4 days. Cells were then incubated for an additional 2 h with CCK-8 reagent (100 μL/mL medium) and read at 450 nm using a microplate reader (Thermo, Varioskan Flash). Results were reproduced in six different wells, and repeated at least three times. Absorbance was monitored and the relative cell viability was calculated. Cell viability rate was expressed as percentage of control samples in each group.

### Western blot analysis

To understand the molecular pathway of VEGF in diabetes-induced testicular damage, RTSCs were lysed with RIPA Lysis Buffer (Santa Cruz Biotechnology, Santa Cruz, CA) containing protease inhibitors (Complete; Roche, Mannheim, Germany) and phosphatase inhibitors (PhosStop; Roche, Mannheim, Germany). The protein concentration in the supernatants with steps according to the manufacturer's protocol was measured by BCA assay (Beyotime Biotechnology, Haimen, China). Total protein (50 μg) was added to each lane onto 10% SDS-polyacrylamide gels. Blots were blocked with 5% nonfat dried milk for 1hr. After incubating overnight at 4°C with the relevant primary antibody, HRP-conjugated secondary antibodies were detected by enhanced chemiluminescence reagent (Millipore Corp., Bedford, MA). GAPDH protein was used to normalize the total tissue lysate on the same membrane. All western blots were done with 1:1,000 antibody dilutions.

## Abbreviations

VEGF: vascular endothelial growth factor
T2DM: type 2 diabetes mellitus
ET: endothelial
PI3K: phosphoinositide 3-kinase
RTSC: rats sertoli cell
ROS: reactive oxygen species
GLUT1: glucose transporter 1
STZ: streptozotocin
HF: high-fat diet

## References

[1] Wild S, Roglic G, Green A, Sicree R, King H (2004). Global prevalence of diabetes: estimates for the year 2000 and projections for 2030. Diabetes Care.

[2] Shaw JE, Sicree RA, Zimmet PZ Global estimates of the prevalence of diabetes for 2010 and 2030. Diabetes Res Clin Pract.

[3] Uysal N, Yalaz G, Acikgoz O, Gonenc S, Kayatekin BM (2005). Effect of L-carnitine on diabetogenic action of streptozotocin in rats. Neuro Endocrinol Lett.

[4] Guneli E, Tugyan K, Ozturk H, Gumustekin M, Cilaker S, Uysal N (2008). Effect of melatonin on testicular damage in streptozotocin-induced diabetes rats. Eur Surg Res.

[5] Aksu I, Baykara B, Kiray M, Gurpinar T, Sisman AR, Ekerbicer N, Tas A, Gokdemir-Yazar O, Uysal N (2013). Serum IGF-1 levels correlate negatively to liver damage in diabetic rats. Biotech Histochem.

[6] Sisman AR, Kiray M, Camsari UM, Evren M, Ates M, Baykara B, Aksu I, Guvendi G, Uysal N (2014). Potential novel biomarkers for diabetic testicular damage in streptozotocin-induced diabetic rats: nerve growth factor beta and vascular endothelial growth factor. Dis Markers.

[7] Maresch CC, Stute DC, Ludlow H, Hammes HP, de Kretser DM, Hedger MP, Linn T (2017). Hyperglycemia is associated with reduced testicular function and activin dysregulation in the Ins2 Akita+/− mouse model of type 1 diabetes. Mol Cell Endocrinol.

[8] Oksanen A (1975). Testicular lesions of streptozotocin diabetic rats. Horm Res.

[9] Sexton WJ, Jarow JP (1997). Effect of diabetes mellitus upon male reproductive function. Urology.

[10] Shrilatha B, Muralidhara (2007). Occurrence of oxidative impairments, response of antioxidant defences and associated biochemical perturbations in male reproductive milieu in the Streptozotocin-diabetic rat. Int J Androl.

[11] Shrilatha B, Muralidhara (2007). Early oxidative stress in testis and epididymal sperm in streptozotocin-induced diabetic mice: its progression and genotoxic consequences. Reprod Toxicol.

[12] Schoeller EL, Albanna G, Frolova AI, Moley KH (2012). Insulin rescues impaired spermatogenesis via the hypothalamic-pituitary-gonadal axis in Akita diabetic mice and restores male fertility. Diabetes.

[13] He X, Li M, Guo F, Xie D (2012). Reduced VEGF signaling in corpus cavernosum of rat with alloxan induced type I diabetes mellitus. Life Scie J.

[14] Ebisch IM, Thomas CM, Wetzels AM, Willemsen WN, Sweep FC, Steegers-Theunissen RP (2008). Review of the role of the plasminogen activator system and vascular endothelial growth factor in subfertility. Fertil Steril.

[15] Ruszkowska-Ciastek B, Sokup A, Socha MW, Ruprecht Z, Halas L, Goralczyk B, Goralczyk K, Gadomska G, Rosc D (2014). A preliminary evaluation of VEGF-A, VEGFR1 and VEGFR2 in patients with well-controlled type 2 diabetes mellitus. J Zhejiang Univ Sci B.

[16] Smith GA, Fearnley GW, Harrison MA, Tomlinson DC, Wheatcroft SB, Ponnambalam S (2015). Vascular endothelial growth factors: multitasking functionality in metabolism, health and disease. J Inherit Metab Dis.

[17] Wu J, Wei H, Qu H, Feng Z, Long J, Ge Q, Deng H (2017). Plasma vascular endothelial growth factor B levels are increased in patients with newly diagnosed type 2 diabetes mellitus and associated with the first phase of glucose-stimulated insulin secretion function of beta-cell. J Endocrinol Invest.

[18] Leung DW, Cachianes G, Kuang WJ, Goeddel DV, Ferrara N (1989). Vascular endothelial growth factor is a secreted angiogenic mitogen. Science.

[19] Ebisch IM, Thomas CM, Wetzels AM, Willemsen WN, Sweep FC, Steegers-Theunissen RP (2008). Review of the role of the plasminogen activator system and vascular endothelial growth factor in subfertility. Fertil Steril.

[20] Byeon SH, Lee SC, Choi SH, Lee HK, Lee JH, Chu YK, Kwon OW (2010). Vascular endothelial growth factor as an autocrine survival factor for retinal pigment epithelial cells under oxidative stress via the VEGF-R2/PI3K/Akt. Invest Ophthalmol Vis Sci.

[21] Takahashi K, Miyokawa-Gorin K, Handa K, Kitahara A, Moriya R, Onuma H, Sumitani Y, Tanaka T, Katsuta H, Nishida S, Yoshimoto K, Ohno H, Ishida H (2013). Endogenous oxidative stress, but not ER stress, induces hypoxia-independent VEGF (120) release through PI3K-dependent pathways in 3T3-L1 adipocytes. Obesity.

[22] Buzzi F, Xu L, Zuellig RA, Boller SB, Spinas GA, Hynx D, Chang Z, Yang Z, Hemmings BA, Tschopp O, Niessen M (2010). Differential effects of protein kinase B/Akt isoforms on glucose homeostasis and islet mass. Mol Cell Biol.

[23] Gibbons CH, Freeman R (2015). Treatment-induced neuropathy of diabetes: an acute, iatrogenic complication of diabetes. Brain.

[24] Tzeng TF, Liu WY, Liou SS, Hong TY, Liu IM (2016). Antioxidant-rich extract from plantaginis semen ameliorates diabetic retinal injury in a streptozotocin-induced diabetic rat model. Nutrients.

[25] Jesmin S, Al Mamun MA, Rahman MA, Islam MM, Sultana SN, Khatun T, Kawano S; HDRCRP (2014). A novel approach to prevent cardiac complication in diabetes through pharmacological intervention by restoring coronary microcirculation and VEGF signaling cascade in diabetes. Diabetes Res Clin Pract.

[26] Reed MJ, Meszaros K, Entes LJ, Claypool MD, Pinkett JG, Gadbois TM, Reaven GM (2000). A new rat model of type 2 diabetes: the fat-fed, streptozotocin-treated rat. Metabolism.

[27] Srinivasan K, Viswanad B, Asrat L, Kaul CL, Ramarao P (2005). Combination of high-fat diet-fed and low-dose streptozotocin-treated rat: a model for type 2 diabetes and pharmacological screening. Pharmacol Res.

[28] Shoveller AK, Stoll B, Ball RO, Burrin DG (2005). Nutritional and functional importance of intestinal sulfur amino acid metabolism. J Nutr.

[29] Long L, Wang J, Lu X, Xu Y, Zheng S, Luo C, Li Y (2015). Protective effects of scutellarin on type II diabetes mellitus-induced testicular damages related to reactive oxygen species/Bcl-2/Bax and reactive oxygen species/microcirculation/staving pathway in diabetic rat. J Diabetes Res.

[30] Caires KC, de Avila J, McLean DJ (2009). Vascular endothelial growth factor regulates germ cell survival during establishment of spermatogenesis in the bovine testis. Reproduction.

[31] Schmidt JA, de Avila JM, McLean DJ (2006). Effect of vascular endothelial growth factor and testis tissue culture on spermatogenesis in bovine ectopic testis tissue xenografts. Biol Reprod.

[32] De Bock K, Georgiadou M, Carmeliet P (2013). Role of endothelial cell metabolism in vessel sprouting. Cell Metab.

[33] De Bock K, Georgiadou M, Schoors S, Kuchnio A, Wong BW, Cantelmo AR, Quaegebeur A, Ghesquiere B, Cauwenberghs S, Eelen G, Phng LK, Betz I, Tembuyser B (2013). Role of PFKFB3-driven glycolysis in vessel sprouting. Cell.

[34] Guzel D, Dursun AD, Ficicilar H, Tekin D, Tanyeli A, Akat F, Topal Celikkan F, Sabuncuoglu B, Bastug M (2016). Effect of intermittent hypoxia on the cardiac HIF-1/VEGF pathway in experimental type 1 diabetes mellitus. Anatol J Cardiol.

[35] Kivela R, Silvennoinen M, Touvra AM, Lehti TM, Kainulainen H, Vihko V (2006). Effects of experimental type 1 diabetes and exercise training on angiogenic gene expression and capillarization in skeletal muscle. FASEB J.

[36] Chen J, Gu Z, Wu M, Yang Y, Zhang J, Ou J, Zuo Z, Wang J, Chen Y (2016). C-reactive protein can upregulate VEGF expression to promote ADSC-induced angiogenesis by activating HIF-1alpha via CD64/PI3k/Akt and MAPK/ERK signaling pathways. Stem Cell Res Ther.

[37] Hou Y, Ryu CH, Jun JA, Kim SM, Jeong CH, Jeun SS (2014). IL-8 enhances the angiogenic potential of human bone marrow mesenchymal stem cells by increasing vascular endothelial growth factor. Cell Biol Int.

[38] Hye Kim J, Gyu Park S, Kim WK, Song SU, Sung JH (2015). Functional regulation of adipose-derived stem cells by PDGF-D. Stem Cells.

[39] Olszewska-Pazdrak B, Carney DH (2013). Systemic administration of thrombin peptide TP508 enhances VEGF-stimulated angiogenesis and attenuates effects of chronic hypoxia. J Vasc Res.

[40] Olszewska-Pazdrak B, Hein TW, Olszewska P, Carney DH (2009). Chronic hypoxia attenuates VEGF signaling and angiogenic responses by downregulation of KDR in human endothelial cells. Am J Physiol Cell Physiol.

[41] Sargent KM, McFee RM, Spuri Gomes R, Cupp AS (2015). Vascular endothelial growth factor A: just one of multiple mechanisms for sex-specific vascular development within the testis?. J Endocrinol.

[42] Marti HH, Risau W (1998). Systemic hypoxia changes the organ-specific distribution of vascular endothelial growth factor and its receptors. Proc Natl Acad Sci U S A.

[43] Tchaikovski V, Olieslagers S, Bohmer FD, Waltenberger J (2009). Diabetes mellitus activates signal transduction pathways resulting in vascular endothelial growth factor resistance of human monocytes. Circulation.

[44] Chen Y, Wu Y, Gan X, Liu K, Lv X, Shen H, Dai G, Xu H (2016). Iridoid glycoside from Cornus officinalis ameliorated diabetes mellitus-induced testicular damage in male rats: involvement of suppression of the AGEs/RAGE/p38 MAPK signaling pathway. J Ethnopharmacol.

[45] Nalbandian A, Dettin L, Dym M, Ravindranath N (2003). Expression of vascular endothelial growth factor receptors during male germ cell differentiation in the mouse. Biol Reprod.

[46] Manning BD, Toker A (2017). AKT/PKB signaling: navigating the network. Cell.

[47] Lee MY, Luciano AK, Ackah E, Rodriguez-Vita J, Bancroft TA, Eichmann A, Simons M, Kyriakides TR, Morales-Ruiz M, Sessa WC (2014). Endothelial Akt1 mediates angiogenesis by phosphorylating multiple angiogenic substrates. Proc Natl Acad Sci U S A.

[48] Ackah E, Yu J, Zoellner S, Iwakiri Y, Skurk C, Shibata R, Ouchi N, Easton RM, Galasso G, Birnbaum MJ, Walsh K, Sessa WC (2005). Akt1/protein kinase Balpha is critical for ischemic and VEGF-mediated angiogenesis. J Clin Invest.

[49] Nakagawa T (2007). Uncoupling of the VEGF-endothelial nitric oxide axis in diabetic nephropathy: an explanation for the paradoxical effects of VEGF in renal disease. Am J Physiol Renal Physiol.

[50] Gutterman DD, Chabowski DS, Kadlec AO, Durand MJ, Freed JK, Ait-Aissa K, Beyer AM (2016). The human microcirculation: regulation of flow and beyond. Circ Res.

[51] Ostergaard L, Granfeldt A, Secher N, Tietze A, Iversen NK, Jensen MS, Andersen KK, Nagenthiraja K, Gutierrez-Lizardi P, Mouridsen K, Jespersen SN, Tonnesen EK (2015). Microcirculatory dysfunction and tissue oxygenation in critical illness. Acta Anaesthesiol Scand.

[52] Moore JP, Dyson A, Singer M, Fraser J (2015). Microcirculatory dysfunction and resuscitation: why, when, and how. Br J Anaesth.

[53] Herrmann J, Kaski JC, Lerman A (2012). Coronary microvascular dysfunction in the clinical setting: from mystery to reality. Eur Heart J.

[54] Petrache I, Petrusca DN, Bowler RP, Kamocki K (2011). Involvement of ceramide in cell death responses in the pulmonary circulation. Proc Am Thorac Soc.

[55] Matsuda N, Hattori Y (2007). Vascular biology in sepsis: pathophysiological and therapeutic significance of vascular dysfunction. J Smooth Muscle Res.

[56] Lotrionte M, Galiuto L, Biondi-Zoccai GG, Abbate A (2006). Pathophysiologic role of myocardial hypertrophy, microcirculatory dysfunction and cardiomyocyte apoptosis in aortic stenosis [Article in Italian]. G Ital Cardiol (Rome).

[57] Makis AC, Hatzimichael EC, Bourantas KL (2000). The role of cytokines in sickle cell disease. Ann Hematol.

[58] Laurence J, Mitra D (1997). Apoptosis of microvascular endothelial cells in the pathophysiology of thrombotic thrombocytopenic purpura/sporadic hemolytic uremic syndrome. Semin Hematol.

[59] Boitrelle F, Guthauser B, Alter L, Bailly M, Bergere M, Wainer R, Vialard F, Albert M, Selva J (2014). High-magnification selection of spermatozoa prior to oocyte injection: confirmed and potential indications. Reprod Biomed Online.

[60] Toshimori K (2009). Dynamics of the mammalian sperm head: modifications and maturation events from spermatogenesis to egg activation. Adv Anat Embryol Cell Biol.

[61] Waisundara VY, Hsu A, Huang D, Tan BK (2008). Scutellaria baicalensis enhances the anti-diabetic activity of metformin in streptozotocin-induced diabetic Wistar rats. Am J Chin Med.

[62] Su Y, Liu W, Ma L, Liu X, Liu Z, Zhu B (2012). Scutellarin inhibits translocation of protein kinase C in diabetic thoracic aorta of the rat. Clin Exp Pharmacol Physiol.

[63] Connor KM, Krah NM, Dennison RJ, Aderman CM, Chen J, Guerin KI, Sapieha P, Stahl A, Willett KL, Smith LE (2009). Quantification of oxygen-induced retinopathy in the mouse: a model of vessel loss, vessel regrowth and pathological angiogenesis. Nat Protoc.

[64] Ruan GX, Kazlauskas A (2012). Axl is essential for VEGF-A-dependent activation of PI3K/Akt. EMBO J.

[65] Wang Y, Zhou Y, Xiao L, Zheng S, Yan N, Chen D (2017). E2f1 mediates high glucose-induced neuronal death in cultured mouse retinal explants. Cell Cycle.

